# Targeting G with TAL Effectors: A Comparison of Activities of TALENs Constructed with NN and NK Repeat Variable Di-Residues

**DOI:** 10.1371/journal.pone.0045383

**Published:** 2012-09-24

**Authors:** Michelle L. Christian, Zachary L. Demorest, Colby G. Starker, Mark J. Osborn, Michael D. Nyquist, Yong Zhang, Daniel F. Carlson, Philip Bradley, Adam J. Bogdanove, Daniel F. Voytas

**Affiliations:** 1 Department of Genetics, Cell Biology & Development and Center for Genome Engineering, University of Minnesota, Minneapolis, Minnesota, United States of America; 2 Department of Animal Science, University of Minnesota, Saint Paul, Minnesota, United States of America; 3 Division of Public Health Sciences, Fred Hutchinson Cancer Research Center, Seattle, Washington, United States of America; 4 Department of Plant Pathology, Iowa State University, Ames, Iowa, United States of America; New England Biolabs, Inc., United States of America

## Abstract

The DNA binding domain of Transcription Activator-Like (TAL) effectors can easily be engineered to have new DNA sequence specificities. Consequently, engineered TAL effector proteins have become important reagents for manipulating genomes *in vivo*. DNA binding by TAL effectors is mediated by arrays of 34 amino acid repeats. In each repeat, one of two amino acids (repeat variable di-residues, RVDs) contacts a base in the DNA target. RVDs with specificity for C, T and A have been described; however, among RVDs that target G, the RVD NN also binds A, and NK is rare among naturally occurring TAL effectors. Here we show that TAL effector nucleases (TALENs) made with NK to specify G have less activity than their NN-containing counterparts: fourteen of fifteen TALEN pairs made with NN showed more activity in a yeast recombination assay than otherwise identical TALENs made with NK. Activity was assayed for three of these TALEN pairs in human cells, and the results paralleled the yeast data. The *in vivo* data is explained by *in vitro* measurements of binding affinity demonstrating that NK-containing TAL effectors have less affinity for targets with G than their NN-containing counterparts. On targets for which G was substituted with A, higher G-specificity was observed for NK-containing TALENs. TALENs with different N- and C-terminal truncations were also tested on targets that differed in the length of the spacer between the two TALEN binding sites. TALENs with C-termini of either 63 or 231 amino acids after the repeat array cleaved targets across a broad range of spacer lengths – from 14 to 33 bp. TALENs with only 18 aa after the repeat array, however, showed a clear optimum for spacers of 13 to 16 bp. The data presented here provide useful guidelines for increasing the specificity and activity of engineered TAL effector proteins.

## Introduction

The ability to target proteins to specific DNA sequences makes it possible to manipulate nucleic acids *in vivo*, for example, by creating artificial transcriptional regulators that alter gene expression or by engineering sequence-specific nucleases that modify genetic loci of interest. Work in the past few years has made it increasingly evident that the protein domain of choice for DNA targeting is the DNA binding domain derived from Transcription Activator-Like (TAL) effectors [1]. TAL effectors are proteins produced by bacterial plant pathogens of the genus *Xanthomonas*, and they are delivered to plant cells during infection where they activate the expression of select target genes and thereby make the plant more prone to bacterial colonization [2]. Targeting of TAL effectors to plant gene promoters is achieved by a simple and elegant mechanism of DNA binding [3], [4]. This mechanism enables the pathogen to rapidly evolve TAL effectors with new DNA sequence specificities, and by extension, it enables scientists to engineer DNA binding domains that recognize novel sites for various genome engineering applications.

DNA binding by TAL effectors is mediated by the central domain of the protein, which comprises approximately 13–28 tandem repeats of a 34 amino acid motif [5]. Amino acid sequences of the repeats are largely invariant with the exception of two residues at positions 12 and 13, called the repeat variable di-residues (RVDs). Each repeat forms two alpha helices that are joined at one end by a short loop that contains the RVD: residue 12 reaches back to interact with one of the alpha helices to stabilize the loop, and residue 13 contacts a specific base within the major groove of DNA [3], [4]. Among the most common RVDs in TAL effectors are HD, NG and NI, which specify the nucleotides C, T and A, respectively; these RVDs are only infrequently associated with other bases [5]. The mechanistic basis for this sequence specificity has been explained by the recently reported structure of two TAL effectors bound to DNA [3], [4]. The most common RVD that specifies G, namely NN, also interacts well with A, and this interaction is achieved by hydrogen-bonding between the second asparagine in the RVD and the N_7_ group on these purines [3], [4], [6].

The lack of specificity for G is one potential drawback in the use of engineered TAL effector proteins for DNA targeting. NK, an RVD only rarely found in nature, also interacts with G [7]. In vitro SELEX data suggested that NK RVDs may have higher specificity for G than NN [7]; however, DNA targeting to a locus in zebrafish was more effective when engineered TAL effector proteins were made using repeats with NN RVDs rather than NK [8]. Two recent studies also indicated that NK TAL effectors have less activity than NN-containing proteins, and further, the RVD NH was shown to provide G-specificity [9], [10]. Clearly there is considerable interest in both understanding and increasing G-specificity of engineered TAL proteins.

Other efforts to adopt TAL effectors for genome engineering have focused on defining the minimal region of the protein required for DNA binding [7], [8], [11], [12]. In the TAL effector PthXo1, for example, the repeat region is flanked by 288 and 295 amino acids at the N- and C-termini, respectively. To delimit the DNA binding domain, different research groups have made and tested various N- and C-terminal truncations. An N-terminal truncation at residue 152 and C-terminal truncations up to 18 amino acids after the repeat domain still allow for effective DNA binding [7], [12]. The structure of TAL effectors bound to DNA is consistent with truncations of this extent still being able to bind DNA [3], [4].

The length of the C-terminus after the TAL effector DNA binding domain has a direct impact on the activity of TAL effector nucleases (TALENs) – fusions of the TAL repeat arrays to the catalytic domain of the FokI endonuclease [11]. FokI functions as a dimer, and DNA cleavage is achieved using two TALENs that bind opposing DNA target sites separated by a spacer. The length of the spacer must allow for efficient FokI dimerization so that DNA cleavage is achieved. In all TALEN architectures tested to date, DNA cleavage is occurs across a broad range of spacer lengths [7], [8], [11], [12], and this variability further motivates defining the minimal DNA binding domain so that TALENs can be engineered with maximal activity over a narrow range of spacer lengths.

Toward improving TAL protein engineering, we compared the activity of TALENs that use repeats with either NN or NK RVDs to target G. We conclude that whereas TALENs made with NK RVDs may have more specificity for G-containing targets, this specificity is offset by a considerable reduction in activity that is due to having less affinity for G than their NN-containing counterparts. We also compared the activity of TALENs with various N- and C-terminal deletions and found a novel architecture that cleaves optimally over a narrower spacer length range than any reported to date.

## Methods

### TALEN construction

TALEN target sites were identified using the TAL effector-Nucleotide Targeter (TALE-NT) program [11]. All targets chosen had a T at the -1 position. TALENs recognizing the target sites were constructed using our previously reported assembly method that is based on Golden Gate cloning [13]. In brief, Golden Gate cloning uses Type IIS restriction endonucleases (e.g. BsaI, Esp3I) to create unique 4 bp overhangs on DNA fragments so that they can be assembled in a precise, sequential order. Our library of plasmids encodes TAL effector repeats with five different RVDs (NI, HD, NN, NG and NK) that can be released by digestion with BsaI to create unique 4 bp ends. This enables up to 10 RVD-encoding plasmids to be ligated in the correct order in a single reaction. Sub-arrays of 10 TAL effector repeats can then be joined simultaneously in a second, similar reaction, resulting in a fully-assembled array (11–31 repeats) cloned upstream a FokI nuclease domain in a yeast expression vector.

The pTAL-BamHI expression vector contains 288 aa in the N-terminus of the repeat array and 231 aa in the C-terminus. Truncations of the pTAL-BamHI backbone were constructed by first PCR amplifying fragments encoding the desired length of N or C-termini, along with the sequences necessary to perform subsequent Golden Gate cloning reactions. These amplicons were then cloned into a linearized pTAL backbone lacking the TALE termini to yield the NΔ152/C+63 and NΔ152/C+18 truncated TALEN plasmids. These plasmids are compatible with the Golden Gate assembly platform.

### Yeast single-strand annealing assay

The yeast-based assay for testing TALEN function has previously been described [13], [14]. Briefly, a yeast strain expressing an engineered TALEN(s) is mated with a yeast strain carrying a corresponding target plasmid. Cleavage of the target and subsequent recombination by single-strand annealing reconstitutes a functional *lacZ* gene and provides a quantitative readout of TALEN activity. β-galactosidase measurements for all data were normalized to the TALEN, SurB, which is highly active in both yeast and at its endogenous target (Y. Zhang and D. Voytas, unpublished). Statistical significance was assessed using a paired t-test. Differences at *p*<0.05 were considered to be statistically significant. For some datasets, a ZFN positive control derived from the Zif268 zinc finger array was also included.

### Mammalian single-strand annealing assay

Activity of TALENs was measured in HEK293 cells using a single-strand annealing assay that reconstitutes a functional luciferase reporter. The template plasmid for the assay (pSSA-1-3) was kindly provided by Dr. David Segal (UC Davis). PCR-based mutagenesis was used to insert TALEN cleavage sites in pSSA-1-3 between the two halves of the luciferase gene.

Twenty-four hours prior to transfection, HEK293 cells were seeded in quadruplicate in a 24-well dish at a density of 200,000 cells/well in DMEM media containing 10% serum. TALEN monomer plasmids at doses of 100 or 200 µg with 25 ng of the SSA reporter target were transfected into the cells using Lipofectamine 2000 (Invitrogen) according to the manufacturer's instructions. As a control for any basal activity generated by the SSA reporter, the reporter was transfected along with pUC19 plasmid DNA instead of the TALEN. In addition, all reactions included 4 ng of the pRL-TK Renilla luciferase plasmid (Promega) to normalize transfection. At twenty-four hours post transfection, the cells were lysed in 150 µl 1× passive lysis buffer (Promega). Lysate luminescence was measured using the Dual-Luciferase® Reporter Assay System (Promega) according to the manufacturer's instructions. The fold activity of a given TALEN was determined by normalizing the firefly luciferase values to Renilla luciferase, and that value was then divided by the value obtained with the SSA reporter alone.

### Expression and purification of recombinant TALE protein

The bacterial expression vector pGEX6P2-TALE was created by ligating a Golden Gate compatible fragment with the NΔ152/C+63 architecture into pGEX6P2 (GE Healthcare). RVD arrays for specific TALE proteins were then cloned in as described above. Expression constructs encoding TAL effector proteins 166, 167 and 312 were then transformed into Rosetta cells and selected on media containing carbenicillin and chloramphenicol. 200 mL cultures were grown to log phase at 37°C before induction for 3 hours with 1 mM IPTG. The cells were pelleted by centrifugation and lysed in GST lysis buffer (25 mM HEPES pH 7.4, 150 mM NaCl, 5 mM MgCl_2_, 130 µM CaCl_2_, 0.5% Triton X-100, 10% glycerol, 1 mM PMSF, 1 µg/mL Leupeptin, 100 nM Aprotinin, 1 µg/mL Pepstatin A). The lysates were treated with RNase A (20 µg/mL) and DNase I (10 U/mL), clarified by centrifugation (21,000× g, 10 minutes) and then loaded onto a column containing equilibrated Glutathione Sepharose (GE Healthcare). The columns were washed with GST lysis buffer and subsequently by cleavage buffer (50 mM Tris-HCl pH 8.0, 1 mM EDTA, 1 mM DTT, 10% glycerol). Elution of untagged purified TALE protein was performed by overnight incubation at 4°C with PreScission protease (GE Healthcare). Purified TALE proteins were separated by electrophoresis and stained with Coomassie to determine the purity of the samples (Fig. S1).

### Electrophoretic mobility shift assay (EMSA)

Double stranded DNA substrates were prepared by annealing fluorescently tagged complementary oligos. Sequences for substrates used were 5′- TGGACACGACTTGAGCTGTCGTCTTCTGCACTCGTAGTGCTGTGATGA for 166, 5′- TGGACATGACTTGAGCTAGTCAGCACCAGGCATCGTAGTGCTGTGCTGA for 167, 5′- TGGACACGACTTGAGCTGGCGAAAGAGTCCACCACCATCGTAGTGCTGTGCTGA for 312 and 5′-TGGACACGACTTGAGCTCGACGCTCAGGCAACCGTAGTGCTGTGCTGA for the scrambled target. The purified proteins were diluted into binding buffer (10 mM HEPES pH 7.6, 10% glycerol, 100 mM KCl, 10 mM MgCl2, 100 µM EDTA, 500 µ M DTT) at varying concentrations with a fixed concentration of the labeled DNA substrate (20 nM). The reactions were incubated for 30 minutes at room temperature and then separated by electrophoresis on a 7% TBE-acrylamide gel. Detection of the labeled substrate was then performed on a fluorescent scanner (Storm 860, Molecular Dynamics).

### Modeling of NN and NK RVD-DNA interactions

To gain insight into potential determinants of affinity and specificity, NN and NK RVDs were modeled onto all RVD loop backbones in the available crystal structures [3], [4]. Corresponding DNA target site positions were mutated sequentially to adenine and guanine, and the energy of the protein-DNA complex was optimized by Monte Carlo sampling of rotameric sidechain conformations together with gradient-based minimization, allowing small shifts to the protein and DNA backbones. Simulations were performed with the Rosetta software package [15].

## Results

### Activity of TALENs with NN versus NK RVDs

**Figure 1:**
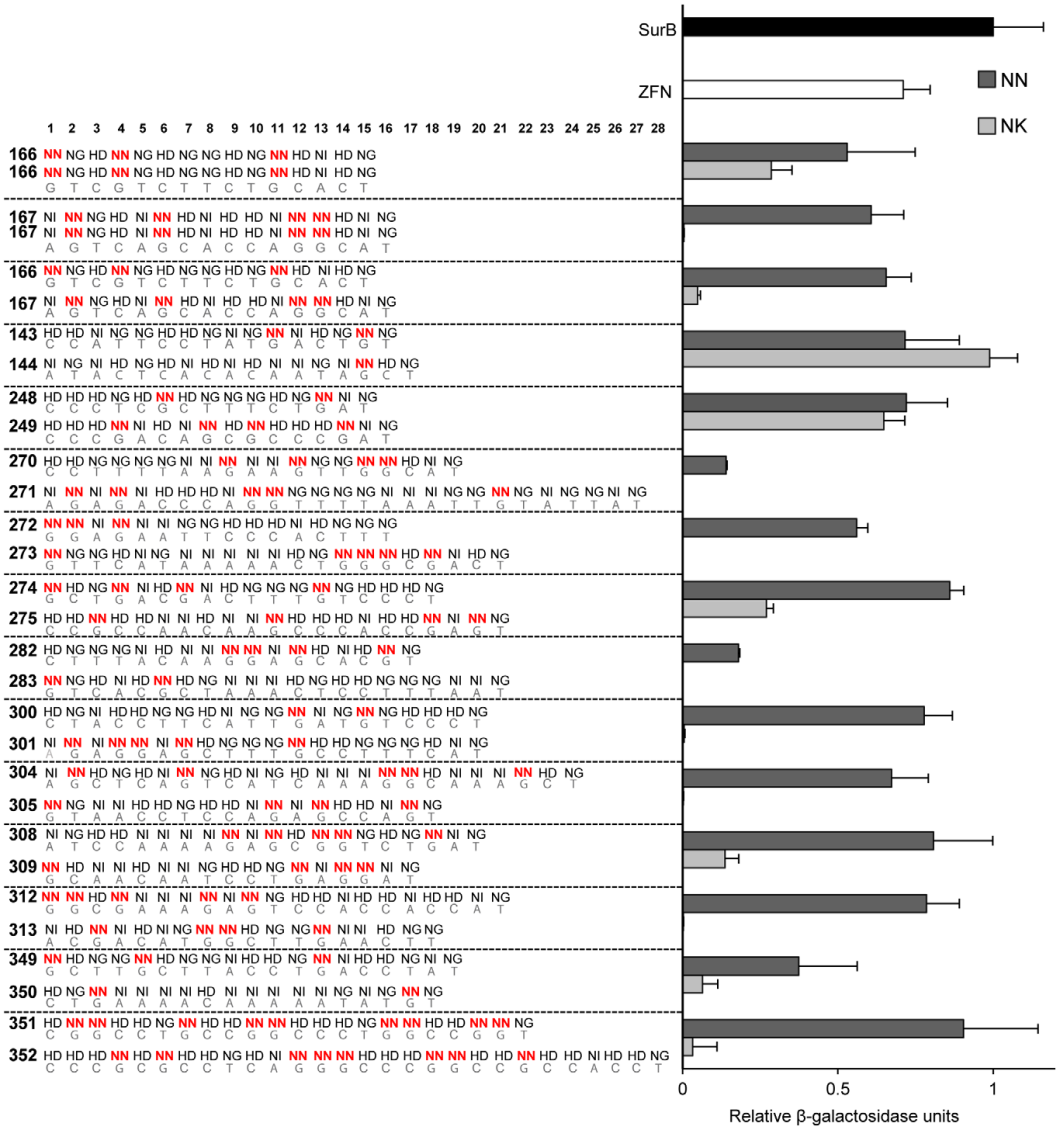
Activity of TALENs containing NN or NK RVDs. Plotted on the right are the relative activities of TALENs with either all NN (dark gray) or NK (light gray) RVDs for specifying G nucleotides. Activities are relative β-galactosidase units as determined in our yeast-based TALEN activity assay [11]. All values are normalized to a TALEN positive control, SurB (black). Also shown for reference is the activity obtained with the well-characterized ZFN, Zif268 (white). Left and right arrays comprising TALEN pairs are numbered (e.g. 166, 167) followed by RVD sequences of the various TALEN DNA binding domains with repeat 1 being nearest to the N-terminus of the protein. NN RVDs that were replaced with NK are in red. The DNA target sequences are shown below the RVDs. Values are expressed as the mean of triplicates with standard deviation (S.D.).

We compared the activities of engineered TALENs that specify the nucleotide G with either NN or NK RVDs. Using the Golden Gate assembly method for TALEN engineering [13], we constructed 15 TALEN pairs that incorporate either NN or separately NK RVDs to specify G. The total number of G's in the corresponding target DNA sequences ranged from 3 to 17, and no bias was imposed as to how the G's were distributed in the target. To test the activity of the TALENs, we used a yeast-based single-strand annealing (SSA) assay in which LacZ activity serves as an indicator of DNA cleavage by TALENs [11],[14]. Briefly, the assay employs a target plasmid containing a *lacZ* reporter gene with an internal sequence duplication disrupted by a TALEN target sequence. Cleavage of the target by the corresponding TALENs results in reconstitution of a functional *lacZ* gene whose expression is measured by standard LacZ enzymatic assays. TALEN pairs containing NN RVDs showed 14–90% activity of the positive control TALEN pair, SurB, which was slightly more active than the potent zinc-finger nuclease (ZFN), Zif268 (Fig. 1). When NN was exchanged for the NK RVD, nuclease activity was reduced for 14 of 15 TALEN pairs, and for several NK-containing TALENs, no activity was detected. One exception was TALEN pair 248/249, which contained 6 NK RVDs in total (2 in TALEN 248 and 4 in 249). The activities of both NN- and NK-versions of this TALEN pair were comparable. Another exception was 143/144 in which the NK version had slightly higher activity than the NN version.

**Figure 2:**
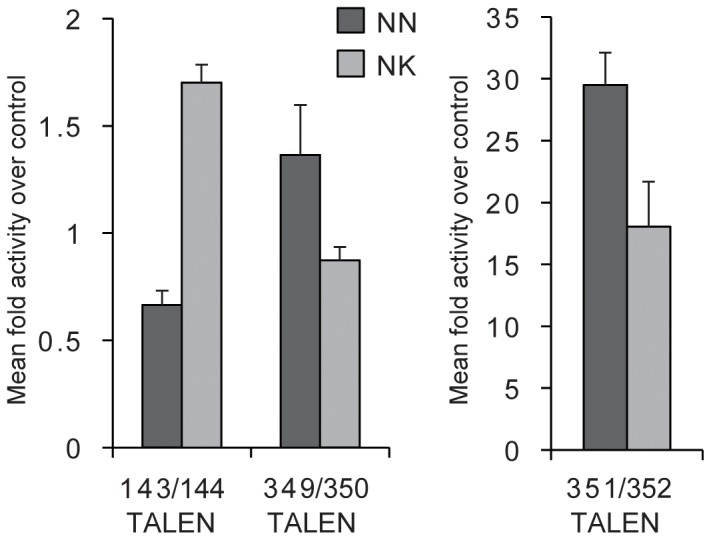
Activity of TALENs containing NN or NK RVDs in human cells. Three TALENs with either all NN (dark gray) or NK (light gray) RVDs were tested in a single-strand annealing (SSA) assay in HEK293 cells. Fold activation levels were determined by normalizing the levels of luciferase activity mediated by TALEN conversion of the SSA luciferase target to the basal level of luciferase exhibited by the SSA plasmid alone. Each treatment group contained a transfection/toxicity control consisting of the Renilla luciferase plasmid. Values are expressed as the mean of quadruplicates with S.D.

We sought to validate the trends observed in yeast by testing several TALEN pairs made with either NN or NK RVDs in human cells. As in yeast, we used a SSA assay in which a luciferase gene is interrupted by an internal sequence duplication and a TALEN recognition site. When the TALEN cleaves the target, the break is repaired, restoring the luciferase open reading frame and thereby allowing for comparison and quantification of TALEN activity. Three target sites were tested, one of which (143/144) was an exception in the yeast assay and showed higher activity for the NK-containing TALEN pair. For all three targets, the activity of TALENs with NN versus NK RVDs recapitulated in human cells what was observed in yeast, although in some cases the magnitude of the difference was considerably less (Fig. 2). For example, with TALEN pair 351/352, the NK version was 30-fold less active in yeast, whereas only 1.6-fold less activity was observed for the NK version in human cells.

### TAL effector arrays containing NK RVDs have lower affinities for DNA targets

**Figure 3:**
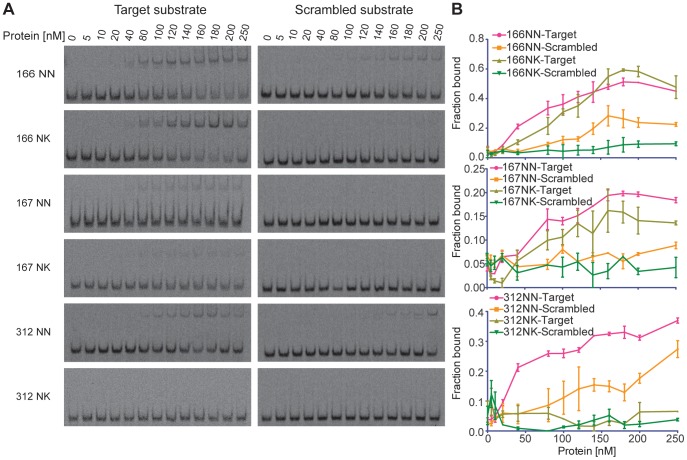
Relative binding affinities of purified TAL effector proteins for DNA targets. (**a**) Representative images of electrophoretic mobility shift assays. Fluorescently-labeled double-stranded oligonucleotide substrates corresponding to either TAL effector targets or a scrambled substrate were incubated with purified TAL effector proteins comprised of either NN or NK RVDs. (**b**) Graphs depicting results from densitometry performed on gel images. Shifted labeled substrate was detected using a fluorescent scanner and quantified by densitometry. The fraction of substrate bound by the TAL effector protein was plotted for each protein concentration. Data indicate the mean ± S.D. for 3 independent experiments.

To understand the basis for the lower activity of NK-containing TALENs *in vivo*, we performed a series of electrophoretic mobility shift assays (EMSAs) using purified TAL effectors 166, 167, and 312, which contain 3, 4, or 5 NN or NK RVDs, respectively. Each NN- or NK-containing array was expressed as a GST fusion protein, purified from *E. coli*, and used to perform EMSAs with cognate targets or a scrambled substrate. All NN-containing arrays bound their target significantly better than the scrambled substrate. For TAL effector 166, both the NN- and NK-containing proteins bound target substrates with similar affinities, as evidenced by the shift of the target probe in the presence of increasing concentration of protein (Fig. 3A). TAL effector 312NN, which contains five NN/NK repeats, showed the most dramatic difference between binding affinities when compared to TAL effector 312NK. A shift of the target probe can be seen for TAL effector 312NN at 40 nM of protein, while there is a lack of visible shift at the highest concentration (250 nM) of TAL effector 312NK. Notably, TAL effector 166NN and 312NN also bound the scrambled substrate with greater affinity than the NK versions. TAL effector 167NN bound its target substrate with higher affinity than the NK-containing array.

The data for all six TAL effector proteins were plotted as the fraction of bound substrate at each protein concentration from 0 nM to 250 nM (Fig. 3B). These data taken together indicate that substituting NK for NN RVDs does contribute to overall lower binding affinities of the TAL effector proteins. Furthermore, the TAL effector protein binding affinities correlate well with the nuclease activities of the corresponding TALENs *in vivo*.

### Effects on TALEN activity of number and position of NK RVDs in repeat arrays

**Figure 4:**
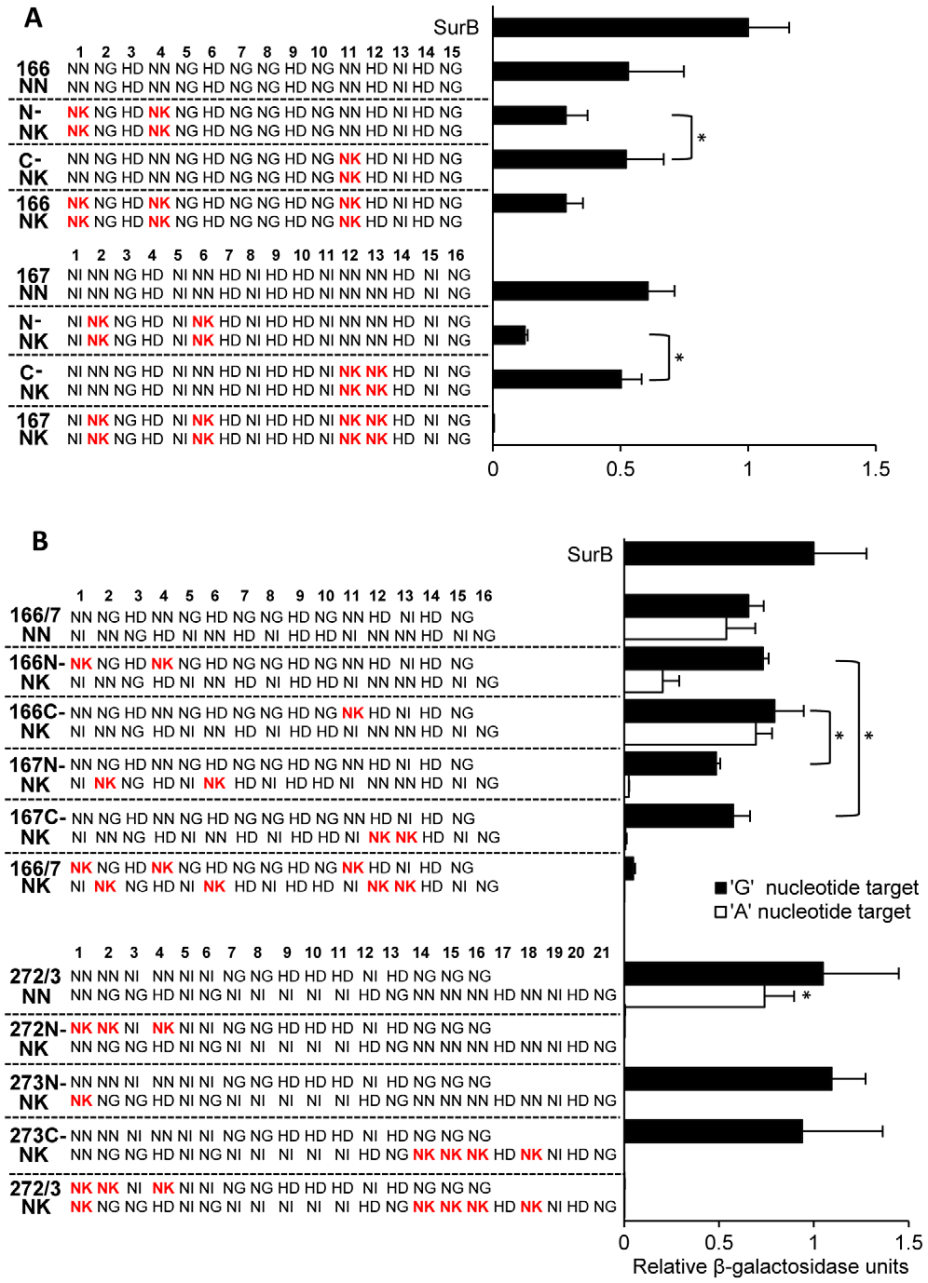
Effects on TALEN activity of number and position of NK RVDs in repeat arrays. (**a**) Activity of homodimeric TALENs. TALEN RVD sequences are shown on the left. Note that the left and right TALENs are identical except for the presence of NN versus NK RVDs. NK RVDs are shown in red for ease of visualization. The activity of the TALEN pairs is plotted on the right. (**b**) Activity of heterodimeric TALENs. TALEN arrays with NK substitutions were tested with TALEN array partners that contain only NN RVDs. Black bars denote activity of TALENs at target sequences with G nucleotides at positions corresponding to NN or NK RVDs. White bars denote TALEN activity with A nucleotides in place of G. All values are normalized to the SurB TALEN positive control. Values are expressed as the mean of quadruplicates with S.D. *, differences in relative β-galactosidase levels between the two indicated samples at *p*<0.05 were considered to be statistically significant.

Having observed predominantly reduced nuclease activity for TALEN pairs with NK versus NN RVDs, we next evaluated the effect on activity of number and/or position of NK RVDs within the array. The Golden Gate TALEN assembly method allows rearrangement of RVDs within existing TALEN pairs with relative ease [13], and so we altered the NN/NK composition of the 166 and 167 TALENs to create four variants of each (Fig. 4A). The 166 and 167 arrays and their variants were then tested as TALENs on homodimeric target sites (i.e. the target site for either the 166 or 167 array was duplicated in inverse orientation and separated by a 15 bp spacer). Two consistent observations were made: 1) as described above, TALENs with NK RVDs had the least activity compared to their NN-containing counterparts, and 2) the most significant impact on activity occurred when the NK RVDs were located within the N-terminal half of the array. This latter observation was particularly pronounced for NK substitutions in the 167 repeat array.

When the 166/167 array variants were tested together as heterodimeric TALENs the impact of the NK RVDs was diminished (Fig. 4B, G-containing targets). For example, when the 166 array variants were tested with a fully NN-containing 167 array, no difference in TALEN activity was observed. Further, the 167-N variant, which showed 5-fold reduction in activity when tested as a homodimer, showed only a modest decrease in activity when tested with the fully NN-containing 166 array. This suggests that NK substitutions that decrease TALEN activity can be ameliorated by pairing with a functional TALEN partner.

NK substitutions were made in repeat arrays for a second pair of TALENs (272/273) (Fig. 4B), and the same general observations were made with respect to the impact of the substitutions on TALEN activity. When both the 272/273 TALEN pairs contained all NK RVDs to specify G, little or no activity was observed. Activity was also compromised when a fully NN-containing TALEN 273 was paired with a TALEN 272 variant with three NK substitutions at the N-terminus. In contrast, four NK substitutions at the C-terminus of a 273 variant had no effect on activity when this TALEN was paired with a fully NN-containing TALEN 272. It appears that the number of NK substitutions also impacts activity, as a single NK substitution at the N-terminus of TALEN 273 did not affect activity when paired with the NN-containing 272 TALEN. We conclude, therefore, that NK substitutions are more likely to compromise TALEN activity when located at the N-terminus of the protein and that the loss of activity is amplified when there are multiple NK substitutions.

### Testing TALENs on G- and A-containing targets

A previous report showed a specificity of NK RVDs for G nucleotides over A nucleotides [7]. In contrast, the NN RVD appears to recognize both G and A [3], [4], [7], [16]. We therefore tested several of our TALEN variants against targets in which G's had been replaced with A's (Fig. 4B, Fig. S2). The fully NN-containing 166/167 and 272/273 TALEN pairs showed comparably high levels of activity on both G- and A-containing targets, whereas little or no activity was observed for the fully NK-containing arrays on both targets. All four 166/167 arrays that varied in the number and distribution of NK RVDs showed higher activity on G-containing targets, and the same was observed for 2 of 3 272/273 TALEN pairs. These data suggest that NK has higher specificity for G over A.

### Modeling of NN and NK RVD-DNA interactions

**Figure 5:**
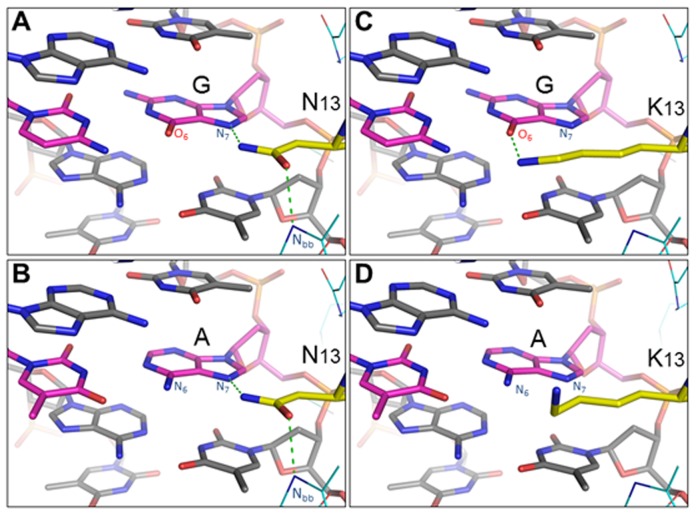
Representative low-energy models of NN:G, NK:G, NN:A, and NK:A interactions. (**a & b**) An NN RVD recognizing either G or A can donate a hydrogen bond to the purine N_7_ atom and accept a hydrogen bond from the backbone nitrogen of residue 13 in the preceding repeat. (**c & d**) When threaded onto the available RVD loop structures, the lysine at position 13 (K13) in an NK RVD is positioned to donate a hydrogen bond to the guanine O_6_ atom, but is unable to participate in the two hydrogen bonds formed by an asparagine (N13) at that position. The NK RVD is not predicted to form hydrogen bonds with adenine.

To assess whether our conclusions regarding the affinity and specificity of NN and NK RVDs for G and A have any structural basis, molecular modeling simulations of potential NN:G, NK:G, NN:A, and NK:A interactions were performed using the two solved TAL-DNA co-complexes as structural templates. As determined from the PthXo1 crystal structure, an NN RVD can donate a hydrogen bond to the N_7_ atom of G or A and accept a hydrogen bond from the backbone nitrogen of residue 13 in the preceding repeat [3] (Fig. 5). When threaded onto the available RVD loop structures, the lysine at position 13 in an NK RVD is positioned to donate a hydrogen bond to the guanine O_6_ atom of guanine, but is unable to participate in the two hydrogen bonds formed by the asparagine at that position in an NN RVD. The potential desolvation of the N_7_ atom, together with the differential loss of sidechain entropy upon binding, may explain the lower affinity of the NK RVD for G relative to the NN:G interaction. In addition, the prediction that NK:G does form a hydrogen bond with guanine and not with adenine may provide the basis for its apparent G-specificity. It should be noted that the NK interactions are theoretical models based on existing TAL crystal structures; large-scale rearrangement of the RVD loop or interacting residues would likely change the set of potential interactions.

### Comparison of TALEN backbone architectures

The first TALEN architecture described contains 288 aa of the N-terminus of Tal1c upstream of the repeat arrays, and 231 aa of the C-terminus downstream of the repeats [11]. This architecture, termed the pTAL-BamHI backbone, has been shown to be active for many TALEN pairs [13]. A second TALEN architecture was described with enhanced nuclease activity, referred to here as the NΔ152/C+63-backbone [7]. The first 152 aa were deleted from the N-terminal portion of the TAL effector protein, upstream of the repeat array (Δ152). A second deletion to the C terminus leaves 63 aa immediately following the last repeat in the repeat array (+63). Other C-terminal truncations of TAL effector proteins have been described [12], and we constructed an additional TALEN backbone with only 18 aa following the half-repeat of the TAL effector array, designated NΔ152/C+63.

**Figure 6:**
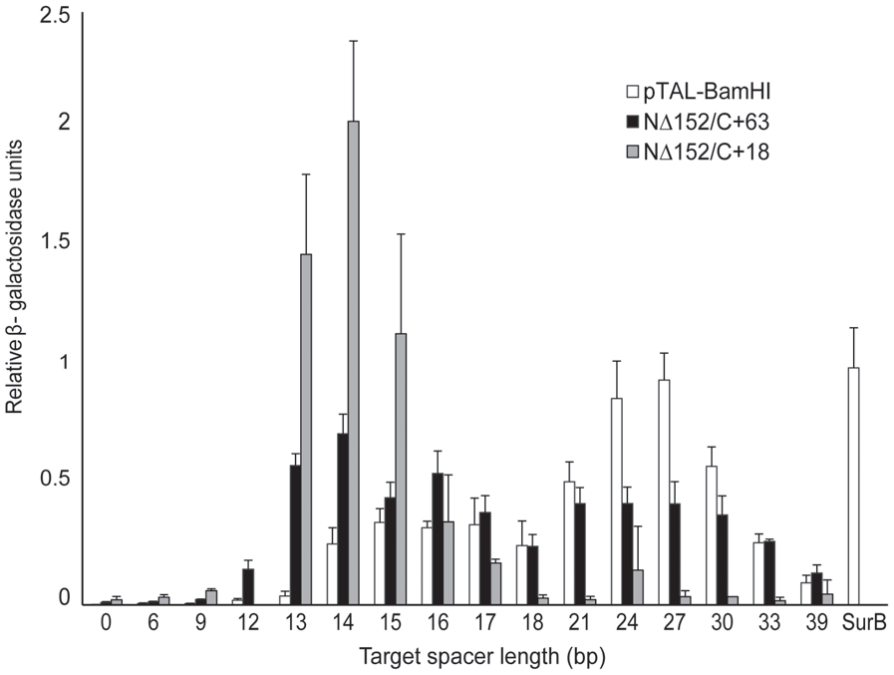
TALEN backbone architectures and spacer length optima. A single TALEN repeat array was placed in either the pTAL-BamHI backbone (white), the NΔ152/C+63 backbone (black) or the NΔ152/C+18 backbone (light gray) and tested against its corresponding palindromic target. The left and right TALENs (23.5 repeats) were separated by spacers of lengths indicated by the values shown along the x-axis. All values are normalized to SurB TALEN positive control. Values are expressed as the mean of quadruplicates with S.D.

To evaluate the relationship between TALEN activity, spacer length and the length of the C-terminus, we cloned a single pair of TAL effector DNA-binding domains in the three architectures described above and tested their activities on targets with spacer lengths varying from 0 to 39 bases. We found that the TALEN pair with the pTAL-BamHI backbone yielded activity that was 25% or greater than the positive control on targets separated by 14–33 bp, with an optimal activity range on spacer lengths of 24–27 bp (Fig. 6). Similarly, the TALEN pair with the NΔ152/C+63-backbone had activity at least 25% of the positive control across spacer lengths ranging from 14–33 bp. In contrast, the NΔ152/C+18 TALEN displayed more focused activity, showing activity exceeding 25% of the positive control for spacer lengths of 13–16 bp. For spacer lengths of 13–15 bp, activities of the NΔ152/C+18 TALENs were 2–3 fold higher than the NΔ152/C+63 versions. We conclude that shorter C-terminal truncations make it possible to achieve optimal TALEN activity over a narrow range of spacer lengths.

## Discussion

Our analysis of the activity of TALENs that target G with either NN or NK RVDs revealed three general conclusions: 1) NN-containing TALENs are more active than their NK-containing counterparts, which can be explained by their overall higher binding affinity for G-containing targets; 2) TALENs made with NK RVDs are more specific for G-containing targets, and 3) NK RVDs have the biggest impact on TALEN activity when they occur in the N-terminal half of the repeat array.

Our study presents, to the best of our knowledge, the first affinity data comparing the effect of RVDs specifying G to the overall affinity of the TAL effector protein. The EMSAs revealed that TAL effectors containing four or five NK RVD substitutions had lower binding affinities than their NN counterparts for the same target. Importantly, the data indicate that the difference in activity of the TALENs can be attributed to the affinity of the TAL effectors for their DNA targets. For example, the NK version of TALEN pair 166 retained over half of the activity of the NN version, which can be explained by the comparable binding affinities of the 166 NN/NK TAL effector arrays *in vitro*. Furthermore, TAL effector 166NN also bound its non-target substrate, indicating it has higher affinity for DNA in general, which could account for the increased nuclease activity observed for TALEN pair 166NN (Fig. 1). The nuclease activities of TALEN pairs 167 and 312 NN/NK also reflect the observed binding affinities of the TAL effector proteins, in that the NK-containing arrays showed both decreased nuclease activity and lower binding affinity.

Our conclusions are consistent with other studies that evaluated the activity of engineered TAL effector proteins with NN and/or NK RVDs. In one study, the activity of a single TALEN pair was evaluated that contained a mixture of both NK and NN RVDs [7]. These TALENs were active and able to introduce mutations in the intended target site at frequencies exceeding 3%. The TALENs making up this pair contained no NK RVDs within the first five repeats of either array, and of the 36 RVDs in both arrays, 5 were NKs (2 in one array and 3 in the other). The observed activity of this TALEN, therefore, is consistent with our finding that weak RVD/nucleotide associations are tolerated if they occur in the C-terminal half of the array. SELEX assays were also performed with four engineered TAL effector proteins to profile base specificity, and consistent with our data, NK was found to be more specific for G than NN.

A second study compared the activity of a TALEN pair targeting an endogenous gene in zebrafish [8]. Both NN and NK versions of the TALEN pair were evaluated, and the NK version was 6-fold less active in mutagenesis than its NN counterpart. Each array of this TALEN pair contained a single NK RVD: one was located in the first repeat of the left array and the other in the seventh repeat of the right. The observed difference in activity of the two TALEN pairs is consistent with our data showing that NK RVDs located in the N-terminal half of the array have the greatest impact on activity.

While this paper was in review, two additional studies appeared with data regarding the activity of TAL effectors targeting the nucleotide G. Both studies presented evidence consistent with our own observations, namely that TAL effectors with NK RVDs had compromised activity in a transcriptional activation assay compared to the corresponding NN-containing proteins [9], [10]. This led to the conclusion that NK is a ‘weak’ RVD and does not contribute significantly to the affinity of the protein, but does retain specificity for G. The authors also suggested that NN is a ‘strong’ RVD and thus contributes more to the overall binding of the TALE. Our EMSA results are consistent with this conclusion, as illustrated by the 166NN TAL effector and TALEN data. Both studies also identified NH as an RVD that targets G with good specificity. It would be interesting to create NH-containing variants of the proteins tested here to directly measure their affinity and compare them to NN- and NK-containing counterparts.

It should also be noted that there is a difference in amino acid composition of the repeats used to make TALENs in this study as compared to the studies mentioned previously. TALENs assembled by our Golden-Gate method have a serine adjacent to the NN RVD (i.e. SNN), whereas previous studies use an NN preceded by asparagine (NNN). Although this difference is unlikely to contribute to the overall activity of the TALENs, the impact of this difference was not addressed experimentally in this study.

The recently reported crystal structures of TAL effectors bound to DNA provides some insight into the activity we observe for TALENs made with NN versus NK RVDs [3], [4]. The N at position 13 in the NN RVD can form a hydrogen bond with N_7_ of either guanine or adenine. For NK, the longer side chain of lysine likely cannot form this bond, and this would negatively impact the affinity of NK-containing repeat arrays for their targets. Additional structures of TAL effectors with NK RVDs should inform this hypothesis and also shed light on differences in how NN and NK RVDs achieve their base specificity.

The crystal structure of the PthXo1TAL effector, which contains 23.5 repeats, showed some disorder at the C-terminus with respect to its interaction with DNA [3]. Whether this disorder reflects a diminished role for the C-terminus in DNA binding remains to be determined; however, it is consistent with our finding that suboptimal RVDs at the C-terminus are better tolerated than those at the N-terminus. The activity of other engineered TAL effector proteins with so-called ‘mismatches’ between RVDs and their intended bases are also consistent with our findings. One study tested the impact of mismatches on transcriptional activation by engineered TAL effector transcriptional activators [17]. Mismatches near the N-terminus of the engineered TAL effectors typically had a greater negative impact on transcriptional activation than those near the C-terminus, and negative effects were more pronounced if two mismatches occurred in a row or there were more than two total mismatches in the array. We recognize that all mismatches are not equivalent due to differences in affinity and specificity of each RVD for its preferred base; further, TALEN cleavage or transcriptional activation by TAL effectors are indirect readouts of protein/DNA interactions. Direct biochemical measurements of TAL effector DNA binding affinity and specificity as reported here will provide further insight into how to optimize the engineering of these proteins for *in vivo* use.

In addition to issues pertaining to targeting the nucleotide G, TALEN specificity may also be compromised by the fact that a broad range of spacer lengths support FokI dimerization and target cleavage. For therapeutic purposes, it would be desirable to have a TALEN pair that only cleaves when both monomers are bound to target sites separated by a fixed spacer length. Other groups have accessed the effective range of spacers lengths over which TALENs can function, and their conclusions have been similar to ours, namely that truncations to the C-terminus narrows the spacer range over which TALENs are optimally active [7], [11], [12], [18]. In this study we completed a side-by-side comparison of three different TAL effector architectures using the same TAL effector DNA binding domains. We observed that TALENs with 63 or 183 amino acids after the repeat array cleave spacer lengths ranging from 12–39 bp at 25% of the activity of our highly active positive control. This contrasts with zinc finger nucleases (ZFNs), which are most active when the range of spacer length is limited to a few base pairs [19]. Our data indicate that shortening the C-terminus to 18 residues after the repeat array considerably narrows the range of spacer lengths that support cleavage with the optimal range spanning only 3 bp. Further definition of the TAL effector DNA binding domain may additionally focus the range of spacer lengths that permit cleavage.

We evaluated two aspects of TAL effector protein engineering, namely the suitability of NN and NK RVDs for targeting the nucleotide G and the impact on TALEN activity of both the length of the spacer and the N- and C-termini on either side of the DNA binding domain. We used our yeast-based TALEN activity assay to quickly and quantitatively evaluate these design parameters. In the future, we hope to complement such *in vivo* studies with additional *in vitro* data on the binding activity of TAL effector proteins. We are optimistic that such an integrated approach will provide a deeper understanding of how these proteins interact with DNA and thereby establish best practices for TAL effector protein design.

## Supporting Information

Figure S1 TALEN target sequences used in G- vs. A-target yeast SSA assays.
(DOCX)

Figure S2 Coomassie stained gel confirming the purity of the purified TALE proteins.
(TIF)

## References

[1] Bogdanove AJ, Voytas DF (2011) TAL effectors: customizable proteins for DNA targeting. Science 333: 1843–1846. 21960622 10.1126/science.1204094

[2] Bogdanove AJ, Schornack S, Lahaye T (2010) TAL effectors: finding plant genes for disease and defense. Current Opinion in Plant Biology 13: 394–401. 20570209 10.1016/j.pbi.2010.04.010

[3] Mak AN, Bradley P, Cernadas RA, Bogdanove AJ, Stoddard BL (2012) The crystal structure of TAL effector PthXo1 bound to its DNA target. Science 335: 716–719. 22223736 10.1126/science.1216211 PMC3427646

[4] Deng D, Yan C, Pan X, Mahfouz M, Wang J, et al (2012) Structural basis for sequence-specific recognition of DNA by TAL effectors. Science 335: 720–723. 22223738 10.1126/science.1215670 PMC3586824

[5] Boch J, Bonas U (2010) *Xanthomonas* AvrBs3 family-type III effectors: discovery and function. Annual Review of Phytopathology 48: 419–436. 10.1146/annurev-phyto-080508-081936 19400638

[6] Mahfouz MM, Li L, Shamimuzzaman M, Wibowo A, Fang X, et al (2011) De novo-engineered transcription activator-like effector (TALE) hybrid nuclease with novel DNA binding specificity creates double-strand breaks. Proc Natl Acad Sci U S A in press, DOI: 10.1073/pnas.1019533108. 10.1073/pnas.1019533108 PMC3038751 21262818

[7] Miller JC, Tan S, Qiao G, Barlow KA, Wang J, et al (2011) A TALE nuclease architecture for efficient genome editing. Nature Biotechnology 29: 143–148. 10.1038/nbt.1755 21179091

[8] Huang P, Xiao A, Zhou M, Zhu Z, Lin S, et al (2011) Heritable gene targeting in zebrafish using customized TALENs. Nature Biotechnology 29: 699–700. 10.1038/nbt.1939 21822242

[9] Cong L, Zhou R, Kuo YC, Cunniff M, Zhang F (2012) Comprehensive interrogation of natural TALE DNA-binding modules and transcriptional repressor domains. Nature communications 3: 968. 10.1038/ncomms1962 PMC3556390 22828628

[10] Streubel J, Blucher C, Landgraf A, Boch J (2012) TAL effector RVD specificities and efficiencies. Nature Biotechnology 30: 593–595. 10.1038/nbt.2304 22781676

[11] Christian M, Cermak T, Doyle EL, Schmidt C, Zhang F, et al (2010) Targeting DNA double-strand breaks with TAL effector nucleases. Genetics 186: 757–761. 20660643 10.1534/genetics.110.120717 PMC2942870

[12] Mussolino C, Morbitzer R, Lutge F, Dannemann N, Lahaye T, et al (2011) A novel TALE nuclease scaffold enables high genome editing activity in combination with low toxicity. Nucleic Acids Research 39: 9283–9293. 21813459 10.1093/nar/gkr597 PMC3241638

[13] Cermak T, Doyle EL, Christian M, Wang L, Zhang Y, et al (2011) Efficient design and assembly of custom TALEN and other TAL effector-based constructs for DNA targeting. Nucleic Acids Research 39: e82. 21493687 10.1093/nar/gkr218 PMC3130291

[14] Townsend JA, Wright DA, Winfrey RJ, Fu F, Maeder ML, et al (2009) High-frequency modification of plant genes using engineered zinc-finger nucleases. Nature 459: 442–445. 19404258 10.1038/nature07845 PMC2743854

[15] Leaver-Fay A, Tyka M, Lewis SM, Lange OF, Thompson J, et al (2011) ROSETTA3: an object-oriented software suite for the simulation and design of macromolecules. Methods in enzymology 487: 545–574. 21187238 10.1016/B978-0-12-381270-4.00019-6 PMC4083816

[16] Boch J, Scholze H, Schornack S, Landgraf A, Hahn S, et al (2009) Breaking the code of DNA binding specificity of TAL-type III effectors. Science 326: 1509–1512. 19933107 10.1126/science.1178811

[17] Zhang F, Cong L, Lodato S, Kosuri S, Church GM, et al (2011) Efficient construction of sequence-specific TAL effectors for modulating mammalian transcription. Nature Biotechnology 29: 149–153. 10.1038/nbt.1775 PMC3084533 21248753

[18] Sun N, Liang J, Abil Z, Zhao H (2012) Optimized TAL effector nucleases (TALENs) for use in treatment of sickle cell disease. Molecular bioSystems 8: 1255–1263. 22301904 10.1039/c2mb05461b

[19] Bibikova M, Carroll D, Segal DJ, Trautman JK, Smith J, et al (2001) Stimulation of homologous recombination through targeted cleavage by chimeric nucleases. Molecular and cellular biology 21: 289–297. 11113203 10.1128/MCB.21.1.289-297.2001 PMC88802

