# Supplementary material for: Targeting G with TAL Effectors: A Comparison of Activities of TALENs Constructed with NN and NK Repeat Variable Di-Residues
Source: PLoS One. 2012 Sep 24;7(9):e45383. doi: 10.1371/journal.pone.0045383 (PMC3454392; doi:10.1371/journal.pone.0045383)
Supplement: Figure S1 — TALEN target sequences used in G- vs. A-target yeast SSA assays. (DOCX) [file pone.0045383.s001.docx]

**Figure S1.** TALEN target sequences used in G- vs. A-target yeast SSA assays.

| **TALEN** | **Target sequence** |
| --- | --- |
| 166 | 5'-GTCGTCTTCTGCACT |
| 166A | 5'-ATCATCTTCTACACT |
| 167 | 5'-AGTCAGCACCAGGCAT |
| 167A | 5'-AATCAACACCAAACAT |
| 272 | 5'-GGAGAATTCCCACTTT |
| 272A | 5'-AAAAAATTCCCACTTT |
| 273 | 5'-GTTCATAAAAACTGGG |
| 273A | 5'-ATTCATAAAAACTAAA |
