# Supplementary figures and images for: Targeting G with TAL Effectors: A Comparison of Activities of TALENs Constructed with NN and NK Repeat Variable Di-Residues
Source: PLoS One. 2012 Sep 24;7(9):e45383. doi: 10.1371/journal.pone.0045383 (PMC3454392; doi:10.1371/journal.pone.0045383)

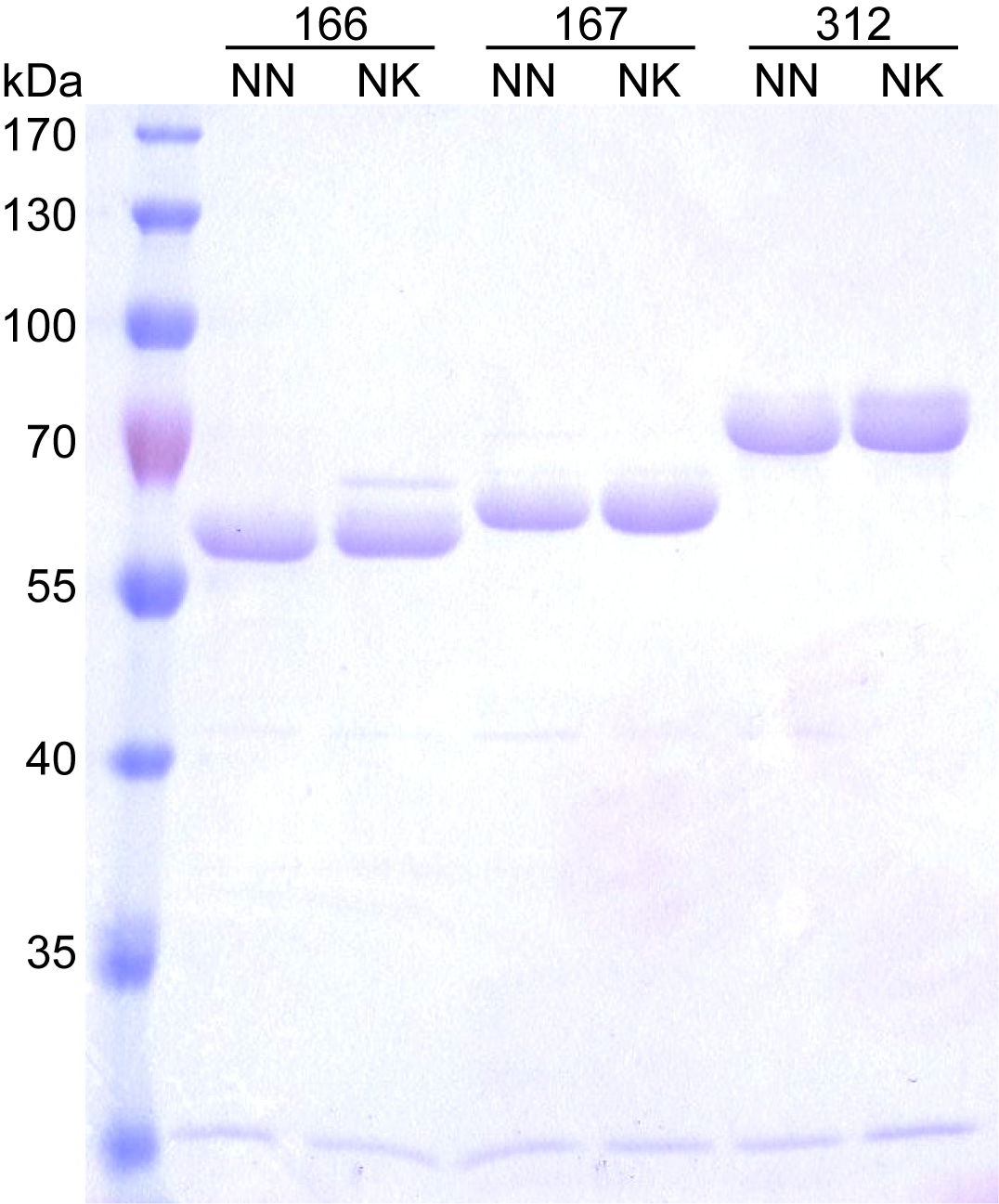

Supplement: Figure S2 — Coomassie stained gel confirming the purity of the purified TALE proteins. (TIF) [file pone.0045383.s002.tif]
